# Absolute risks of self-harm and interpersonal violence by diagnostic category following first discharge from inpatient psychiatric care

**DOI:** 10.1192/j.eurpsy.2022.2352

**Published:** 2023-01-18

**Authors:** P. L. H. Mok, F. Walter, M. J. Carr, S. Antonsen, N. Kapur, S. Steeg, J. Shaw, C. B. Pedersen, R. T. Webb

**Affiliations:** 1 Centre for Pharmacoepidemiology and Drug Safety, Division of Pharmacy & Optometry, The University of Manchester, Manchester, United Kingdom; 2 Manchester Academic Health Science Centre (MAHSC), Manchester, United Kingdom; 3 Division of Nursing, Midwifery and Social Work, The University of Manchester, Manchester, United Kingdom; 4 National Institute for Health and Care Research (NIHR) Greater Manchester Patient Safety Translational Research Centre, The University of Manchester, Manchester, United Kingdom; 5 Centre for Integrated Register-Based Research, Aarhus University, Aarhus, Denmark; 6 National Centre for Register-based Research, Business and Social Sciences, Aarhus University, Aarhus, Denmark; 7 Centre for Mental Health and Safety, Division of Psychology & Mental Health, The University of Manchester, Manchester, United Kingdom

**Keywords:** Discharge from inpatient care, interpersonal violence, psychiatric illness, self-harm, substance misuse

## Abstract

**Background:**

Persons discharged from inpatient psychiatric services are at greatly elevated risk of harming themselves or inflicting violence on others, but no studies have reported gender-specific absolute risks for these two outcomes across the spectrum of psychiatric diagnoses. We aimed to estimate absolute risks for self-harm and interpersonal violence post-discharge according to gender and diagnostic category.

**Methods:**

Danish national registry data were utilized to investigate 62,922 discharged inpatients, born 1967–2000. An age and gender matched cohort study was conducted to examine risks for self-harm and interpersonal violence at 1 year and at 10 years post-discharge. Absolute risks were estimated as cumulative incidence percentage values.

**Results:**

Patients diagnosed with substance misuse disorders were at especially elevated risk, with the absolute risks for either self-harm or interpersonal violence being 15.6% (95% CI 14.9, 16.3%) of males and 16.8% (15.6, 18.1%) of females at 1 year post-discharge, rising to 45.7% (44.5, 46.8%) and 39.0% (37.1, 40.8%), respectively, within 10 years. Diagnoses of personality disorders and early onset behavioral and emotional disorders were also associated with particularly high absolute risks, whilst risks linked with schizophrenia and related disorders, mood disorders, and anxiety/somatoform disorders, were considerably lower.

**Conclusions:**

Patients diagnosed with substance misuse disorders, personality disorders and early onset behavioral and emotional disorders are at especially high risk for internally and externally directed violence. It is crucial, however, that these already marginalized individuals are not further stigmatized. Enhanced care at discharge and during the challenging transition back to life in the community is needed.

## Introduction

Patients discharged from inpatient psychiatric services are at elevated risk for an array of adverse outcomes, such as suicide and other causes of premature mortality, nonfatal self-harm and interpersonal violence perpetration and victimization [1–6]. Previous studies have predominantly examined risks for a single adverse outcome, with some recently published papers reporting risks for multiple adverse outcomes in the same study cohort [5]. Physical harm inflicted on oneself and on other people occurs much more frequently among individuals who have received inpatient psychiatric treatment than in those without such a history [6–10]. However, no study to date has estimated absolute risks for the two outcomes stratified by psychiatric diagnosis in a single cohort of discharged persons, enabling the identification of individuals at especially heightened risk. Self-harm is a major risk factor for suicide [11], and therefore understanding self-harm risks by gender and by diagnostic group among patients discharged from inpatient psychiatric care offers opportunities for tailoring of timely preventive measures. Interpersonal violence causes physical and emotional harm to others and oneself and is also associated with increased risk of dying from unnatural causes [12]. Identifying individuals at higher risk of perpetrating interpersonal violence following discharge from inpatient psychiatric provides further possibilities for preventive follow-up in a patient group likely to be in ongoing contact with mental health services, general healthcare and criminal justice agencies.

In this register-based study, we aimed to compare absolute risks for fatal and nonfatal self-harm and interpersonal violence in a cohort of Danish persons discharged from their first inpatient psychiatric care episode, and to stratify these risks by gender across the full spectrum of psychiatric diagnostic categories. We estimated absolute risks for these adverse outcomes within 1 year and at 10 years post-discharge. This work expands on previously conducted studies in the same study cohort [4, 5]. We hypothesized that risks for self-harm and interpersonal violence perpetration would be higher among discharged patients diagnosed with substance misuse disorders compared to individuals in other diagnostic groups. This is the first study to report on gender-specific absolute risks of the two adverse outcomes across the spectrum of diagnosed mental illnesses, and also the first to report the absolute risk for either outcome occurring post-discharge.

## Methods

### Study cohort

The cohort was delineated from the Danish Civil Registration System [13], which was established in 1968 and records demographics and continuously updated vital status of all Danish residents. The unique identification number assigned to each resident enables accurate linkage with other Danish national registers. Cohort members were all persons born in Denmark during 1967–2000, alive and residing in the country at their 15th birthdays, and with two Danish-born parents. The Data Protection Agency approved the study, with data access agreed by the Health Data Authority and Statistics Denmark. Because the study was conducted using registry data, according to Danish legislation informed consent from cohort members was not required.

### Exposures

Information on first discharge from inpatient psychiatric services was obtained from the Psychiatric Central Research Register [14]. This source was computerized in 1969 and contains data on all admissions to inpatient psychiatric facilities. The following diagnostic categories were examined: substance misuse disorders; schizophrenia and related disorders; mood disorders; anxiety and somatoform disorders; personality disorders; early onset behavioral and emotional disorders; comorbid substance misuse disorder with each of the other diagnostic categories examined; all diagnostic categories combined. The ICD codes used to classify these categories are shown in eTable 1 in the Supplementary Material [15]. Individuals with more than one psychiatric disorder were included in the analysis for each specific disorder, and thus the categories were not mutually exclusive. We also investigated number of diagnostic categories at first discharge (1, 2, 3, or more). Any psychiatric diagnoses not under the six aforementioned categories were grouped together and counted as an additional category.

### Outcomes

#### Self-harm

For consistency with the “Interpersonal violence” outcome (which included homicides), deaths by suicide and nonfatal self-harm episodes were both included. Hospital-presenting self-harm episodes were ascertained using a previously developed algorithm [16], with at least one of the following criteria met according to information recorded in either the National Patient Register [17] or the Psychiatric Central Research Register [14]: (a) Reason for contact code = 4 (National Patient Register); (b) Any psychiatric diagnosis (ICD-10 Chapter F) and a comorbid diagnosis of poisoning with medication and biological compounds (ICD-10 codes T36–T50) or nonmedical compounds, excluding alcohol and poisoning from food (ICD-10 codes T52–T60); (c) Any psychiatric disorder (ICD-10 Chapter F) and comorbid diagnosis reflecting lesions on forearm, wrist or hand (ICD-10 codes S51, S55; S59, S61, S65, or S69); (d) Any hospital contact due to poisoning with weak or strong analgesics, hypnotics, sedatives psychoactive drugs, anti-epileptics and anti-Parkinson drugs or carbon monoxide (ICD-10 codes T39, T42, T43, and T58); (e) Intentional self-harm: ICD-10 X60–X84 (recorded as a primary or secondary diagnosis in either Register). Persons who died by suicide were identified from the Register of Causes of Death [18], classified as ICD-8 E950–E959 or ICD-10 X60–X84, Y87.0.

#### Interpersonal violence

Cases were identified from the National Crime Register [19] and included convictions for homicide, assault, robbery, aggravated burglary or arson, possessing a weapon in a public place, threats of violence, extortion, human trafficking, abduction, kidnapping, rioting and serious public order offenses, terrorism, and sexual offenses. We considered the first violent crime that was committed after 15th birthday—the age when adult criminal responsibility commences in Denmark.

### Study design and analyses

We delineated a matched cohort study that consisted of 62,922 persons aged 15 years and older who had been discharged from inpatient psychiatric care for the first time. Individuals who had been discharged before their 15th birthdays, and those admitted to psychiatric emergency rooms without transfer to an inpatient ward, were excluded. From competing risk survival analyses, gender-specific cumulative incidence percentages (absolute risks) for each adverse outcome were calculated for each diagnostic category separately, within 1 year and at 10 years after the index discharge. For the investigation of risks of adverse outcomes by number of diagnostic categories at first discharge, cumulative incidence plots were generated using Epanechnikov kernel-weighted local polynomial smoothing method to mask step changes associated with small event counts, in accordance with Danish data protection law. Each of the 62,922 discharged patients was matched on date of birth (±1 day) and gender with 25 comparators (*n* = 1,573,050) who were alive, residing in Denmark, and had not been admitted to inpatient psychiatric services on or before the date of first discharge for the person to whom each comparator was matched (henceforth referred to as the “index date”). The discharged cohort and the matched comparators were followed up from the index date until the outcome of interest, death, emigration from Denmark, or December 31, 2015, whichever came first. Analyses were conducted using Stata v15.1.

## Results

### Characteristics of the study cohort

Of the 62,922 individuals aged 15 or over who had been discharged from inpatient psychiatric services for the first time, 51.0% were females. Males were most likely to be diagnosed with substance misuse disorders (Table 1; 33.9% of male patients) and anxiety and somatoform disorders (33.2%), with the most common diagnostic categories for females being anxiety disorders (39.7% of female patients) and mood disorders (30.9%). A diagnosis of an early onset behavioral and emotional disorder at first discharge was relatively rare in this cohort, with 5.6% of males and 4.4% of females classified in this diagnostic group.

**Table 1.** Total numbers of discharged patients by gender and psychiatric diagnostic category.

	Males		Females	
Diagnostic categories^a^	*N*	%	*N*	%
Substance misuse disorders	10,447	33.9	3,466	10.8
Schizophrenia and related disorder	6,053	19.7	4,066	12.7
Mood disorders	6,197	20.1	9,917	30.9
Anxiety and somatoform disorders	10,232	33.2	12,748	39.7
Personality disorders	3,268	10.6	5,686	17.7
Early onset behavioral and emotional disorders	1,737	5.6	1,415	4.4
All discharged patients	30,805	100	32,117	100
Matched general population comparison cohort	770,125	–	802,925	–

a Categories are not mutually exclusive.

### Self-harm risk

Table 2 shows gender-specific cumulative incidence for self-harm at 1 year and 10 years after first discharge from inpatient psychiatric services. Overall, absolute risk of self-harm for males was 8.0% (95% CI 7.7, 8.3%) within 1 year and 22.3% (21.7, 22.8%) within 10 years. Risks were highest among those diagnosed with substance misuse disorders: at 10.4% (9.8, 11.0%) within 1 year, rising to 29.7% (28.7, 30.8%) within 10 years post-discharge. Risks were also particularly high among males diagnosed with a personality disorder: at 10.1% (9.1, 11.2%) within 1 year and 26.4% (24.8, 28.1%) within 10 years post-discharge. Self-harm risk among females was 10.8% (10.5, 11.1%) by 1 year post-discharge and 24.2% (23.7, 24.8%) within 10 years. Females with a diagnosis of early onset behavioral and emotional disorders, substance misuse disorders, and personality disorders at first discharge from inpatient psychiatric care had the highest self-harm risks. Around one in seven of these patients at 1 year, and a third at 10 years, will have had at least one secondary care treated self-harm episode or died by suicide.

**Table 2.** Absolute risk of self-harm within 1 year and at 10 years after first discharge.

	1 year post-discharge:		10 years post-discharge:	
Diagnostic categories	*n*	Cumulative incidence, % (95% CI)	*n*	Cumulative incidence, % (95% CI)
*Males:*				
Substance misuse disorders	1,059	10.4 (9.8, 11.0)	2,486	29.7 (28.7, 30.8)
Schizophrenia and related disorder	324	5.5 (4.9, 6.1)	950	19.3 (18.1, 20.4)
Mood disorders	451	7.5 (6.8, 8.1)	923	18.9 (17.8, 20.1)
Anxiety and somatoform disorders	866	8.7 (8.2, 9.2)	1,712	21.4 (20.4, 22.3)
Personality disorders	328	10.1 (9.1, 11.2)	758	26.4 (24.8, 28.1)
Early onset behavioral and emotional disorders	120	7.2 (6.0, 8.5)	274	21.9 (19.4, 24.6)
All discharged patients	2,411	8.0 (7.7, 8.3)	5,504	22.3 (21.7, 22.8)
Matched general population comparison cohort	1,227	0.17 (0.16, 0.18)	7,675	1.4 (1.4, 1.5)
*Females:*				
Substance misuse disorders	510	15.0 (13.9, 16.3)	1,005	34.2 (32.4, 36.1)
Schizophrenia and related disorder	398	10.0 (9.1, 11.0)	840	24.3 (22.8, 25.8)
Mood disorders	971	10.0 (9.5, 10.6)	1,778	21.4 (20.5, 22.3)
Anxiety and somatoform disorders	1,323	10.6 (10.1, 11.2)	2,443	23.2 (22.4, 24.1)
Personality disorders	813	14.5 (13.6, 15.5)	1,565	31.4 (30.1, 32.7)
Early onset behavioral and emotional disorders	205	15.1 (13.3, 17.1)	379	34.8 (31.7, 38.0)
All discharged patients	3,387	10.8 (10.5, 11.1)	6,549	24.2 (23.7, 24.8)
Matched general population comparison cohort	1,703	0.22 (0.21, 0.23)	8,537	1.5 (1.4, 1.5)

### Interpersonal violence risk

The absolute risk of perpetrating interpersonal violence among males was 4.2% (3.9, 4.4%) within a year of being discharged and around one in six (17.9%; 17.4, 18.4%) at 10 years post-discharge (Table 3). In addition to substance misuse disorders, discharged males with early onset behavioral and emotional disorders also had particularly elevated absolute risks; around a quarter of males in each of these diagnostic groups will have engaged in interpersonal violence at 10 years after first discharge from inpatient psychiatric services. On the contrary, absolute risk was relatively low for males diagnosed with mood disorders (9.1%; 8.2, 10.1%) and schizophrenia or related disorders (13.5%; 12.6, 14.5%) within 10 years of discharge.

**Table 3.** Absolute risk of interpersonal violence perpetration within 1 year and at 10 years after first discharge.

Diagnostic categories	1 year post-discharge:		10 years post-discharge:	
	*n*	Cumulative incidence, % (95% CI)	*n*	Cumulative incidence, % (95% CI)
*Males:*				
Substance misuse disorders	646	6.4 (5.9, 6.9)	2,158	26.7 (25.7, 27.7)
Schizophrenia and related disorder	163	2.8 (2.4, 3.2)	658	13.5 (12.6, 14.5)
Mood disorders	124	2.1 (1.7, 2.5)	388	9.1 (8.2, 10.1)
Anxiety and somatoform disorders	389	4.0 (3.6, 4.4)	1,177	16.1 (15.2, 17.0)
Personality disorders	185	5.8 (5.0, 6.6)	668	23.9 (22.3, 25.5)
Early onset behavioral and emotional disorders	117	7.1 (5.9, 8.4)	318	27.3 (24.3, 30.3)
All discharged patients	1,244	4.2 (3.9, 4.4)	4,198	17.9 (17.4, 18.4)
Matched general population comparison cohort	3,688	0.50 (0.48, 0.51)	16,860	3.0 (2.9, 3.0)
*Females:*				
Substance misuse disorders	78	2.3 (1.8, 2.9)	290	11.1 (9.9, 12.4)
Schizophrenia and related disorder	21	0.53 (0.34, 0.80)	89	2.9 (2.3, 3.6)
Mood disorders	20	0.21 (0.13, 0.31)	110	1.6 (1.3, 1.9)
Anxiety and somatoform disorders	86	0.70 (0.57, 0.86)	309	3.4 (3.0, 3.8)
Personality disorders	51	0.92 (0.69, 1.20)	213	4.7 (4.1, 5.3)
Early onset behavioral and emotional disorders	24	1.8 (1.2, 2.6)	64	6.2 (4.8, 7.9)
All discharged patients	213	0.68 (0.60, 0.78)	847	3.5 (3.3, 3.8)
Matched general population comparison cohort	370	0.048 (0.044, 0.054)	1,814	0.32 (0.30, 0.33)

Whereas absolute risks of self-harm were slightly higher for females versus males, absolute risks of interpersonal violence were much lower among females: 0.68% (0.60, 0.78%) within a year and 3.5% (3.3, 3.8%) at 10 years post-discharge (Table 3). Females diagnosed with substance misuse disorders had the highest risk of interpersonal violence: 11.1% (9.9, 12.4%) at 10 years post-discharge, followed by those diagnosed with early onset behavioral and emotional disorders: 6.2% (4.8, 7.9%) at 10 years. As with males, interpersonal violence risk among women diagnosed with mood disorders (1.6%; 1.3, 1.9%) and schizophrenia or related disorders (2.9%; 2.3, 3.6%) was relatively low at 10 years post-discharge.

### Combined risk of either adverse outcome occurring

In Table 4, cumulative incidence values for either of the two adverse outcomes occurring at 1 year and 10 years post-discharge are presented. A third of males and a quarter of females will have at least one nonfatal or fatal self-harm episode or will have engaged in interpersonal violence within 10 years of their first discharge from inpatient psychiatric care. Risks of one or both adverse outcomes occurring within 10 years after discharge were particularly high among patients diagnosed with substance misuse disorders (45.7% males and 39.0% females), early onset behavioral and emotional disorders (40.1% males and 36.8% females), or personality disorders (40.7% males and 33.3% females).

**Table 4.** Absolute risk of either adverse outcome (self-harm or interpersonal violence) within 1 year and at 10 years after first discharge.

Diagnostic categories	1 year post-discharge:		10 years post-discharge:	
	*n*	Cumulative incidence, % (95% CI)	*n*	Cumulative incidence, % (95% CI)
*Males:*				
Substance misuse disorders	1,593	15.6 (14.9, 16.3)	3,852	45.7 (44.5–46.8)
Schizophrenia and related disorder	470	7.9 (7.3, 8.6)	1,396	28.0 (26.8–29.3)
Mood disorders	556	9.2 (8.5, 10.0)	1,183	24.8 (23.4–26.1)
Anxiety and somatoform disorders	1,189	12.0 (11.3, 12.6)	2,509	31.7 (30.6–32.9)
Personality disorders	492	15.2 (14.0, 16.5)	1,171	40.7 (38.8–42.6)
Early onset behavioral and emotional disorders	224	13.5 (11.9, 15.2)	501	40.1 (36.8–43.3)
All discharged patients	3,475	11.6 (11.2, 11.9)	8,291	33.5 (32.9–34.2)
Matched general population comparison cohort	4,851	0.65 (0.64, 0.67)	22,942	4.1 (4.0–4.1)
*Females:*				
Substance misuse disorders	570	16.8 (15.6, 18.1)	1,140	39.0 (37.1, 40.8)
Schizophrenia and related disorder	414	10.4 (9.5, 11.4)	890	25.9 (24.4, 27.4)
Mood disorders	986	10.2 (9.6, 10.8)	1,829	22.0 (21.1, 23.0)
Anxiety and somatoform disorders	1,391	11.2 (10.7, 11.7)	2,599	24.8 (23.9, 25.6)
Personality disorders	849	15.2 (14.3, 16.1)	1,659	33.3 (31.9, 34.6)
Early onset behavioral and emotional disorders	224	16.5 (14.6, 18.5)	405	36.8 (33.6, 40.0)
All discharged patients	3,547	11.3 (11.0, 11.7)	6,953	25.8 (25.2, 26.3)
Matched general population comparison cohort	2,059	0.27 (0.26, 0.28)	10,022	1.7 (1.7, 1.8)

### Risks associated with substance misuse disorder comorbidity

Table 5 shows the cumulative incidence values for self-harm, interpersonal violence, and for either of these two adverse outcomes at 10 years post-discharge associated with a substance misuse disorder diagnosis in conjunction with one of the other diagnostic categories. Because of the relatively small number of these patients with the adverse outcomes examined at 1 year post-discharge, cumulative incidence values at this follow-up are not reported. Around 3 in 10 males and 1 in 10 females with early onset behavioral and emotional disorders or personality disorders also had a diagnosis of substance misuse disorders. These comorbid diagnostic categories were associated with particularly high absolute risks for the two adverse outcomes investigated. Around half of males and females with a diagnosis of substance misuse in conjunction with personality disorders or early onset behavioral and emotional disorders will self-harm or engage in interpersonal violence within 10 years post-discharge.

**Table 5.** Absolute risk of self-harm, interpersonal violence, and either of the two adverse outcomes, within 10 years after first discharge for patients with co-morbid substance misuse disorders.

Diagnostic categories with co-morbid substance misuse disorders	Number of patients diagnosed (% of all discharged patients; % of patients within the diagnostic category^a^	Self-harm:		Interpersonal violence:		Either adverse outcome	
		*n*	Cumulative incidence, % (95% CI)	*n*	Cumulative incidence, % (95% CI)	*n*	Cumulative incidence, % (95% CI)
*Males:*							
Schizophrenia and related disorder	1,125 (3.7; 18.6)	276	31.3 (28.0, 34.5)	230	27.2 (24.1, 30.4)	422	47.9 (44.2, 51.4)
Mood disorders	1,007 (3.3; 16.2)	233	28.6 (25.3, 31.9)	104	14.5 (11.8, 17.5)	295	36.9 (33.1, 40.6)
Anxiety and somatoform disorders	2,044 (6.6; 20.0)	456	29.4 (26.9, 31.9)	364	24.6 (22.2, 27.1)	689	44.7 (41.9, 47.5)
Personality disorders	926 (3.0; 28.3)	285	35.5 (32.1, 38.9)	285	35.5 (32.1, 38.9)	447	54.6 (50.9, 58.1)
Early onset behavioral and emotional disorders	545 (1.8; 31.4)	104	29.5 (22.8, 36.4)	122	37.2 (28.8, 45.6)	185	53.4 (43.6, 62.2)
All discharged patients	30,805	5,504	22.3 (21.7, 22.8)	4,198	17.9 (17.4, 18.4)	8,291	33.5 (32.9, 34.2)
Matched general population comparison cohort	770,125	7,675	1.4 (1.4, 1.5)	16,860	3.0 (2.9, 3.0)	22,942	4.1 (4.0, 4.1)
*Females:*							
Schizophrenia and related disorder	230 (0.7; 5.7)	67	34.9 (27.7, 42.2)	19	11.6 (7.1, 17.4)	75	39.8 (32.1, 47.3)
Mood disorders	530 (1.7; 5.3)	147	33.3 (28.4, 38.2)	25	7.0 (4.5, 10.3)	158	36.0 (30.9, 41.0)
Anxiety and somatoform disorders	915 (2.8; 7.2)	253	32.8 (29.2, 36.4)	58	9.4 (7.1, 12.1)	281	36.9 (33.1, 40.6)
Personality disorders	584 (1.8; 10.3)	206	40.6 (36.1, 45.0)	55	12.9 (9.8, 16.3)	231	46.2 (41.5, 50.7)
Early onset behavioral and emotional disorders	149 (0.5; 10.5)	38	31.9 (22.5, 41.6)	16	22.2 (11.0, 35.7)	47	43.2 (30.6, 55.0)
All discharged patients	32,117	6,549	24.2 (23.7, 24.8)	847	3.5 (3.3, 3.8)	6,953	25.8 (25.2, 26.3)
Matched general population comparison cohort	802,925	8,537	1.5 (1.4, 1.5)	1,814	0.32 (0.30, 0.33)	10,022	1.7 (1.7, 1.8)

a “Percentage of patients within the diagnostic category” is, for instance, the percentage of all male patients in the schizophrenia and related disorder category who were also diagnosed with a substance misuse disorder at first discharge.

### Risks associated with number of diagnostic categories at first discharge

**Figure 1.**
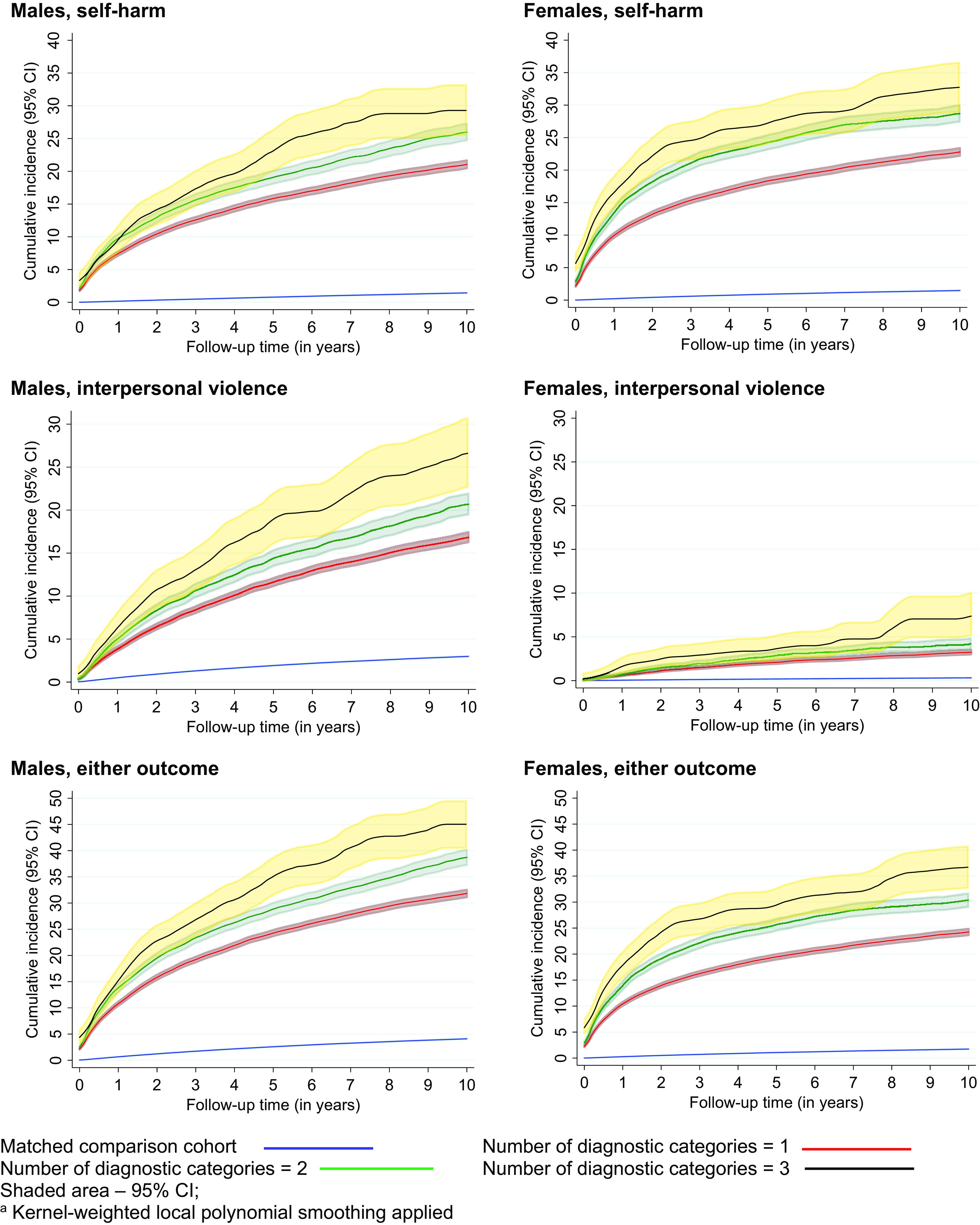
Absolute risks of self-harm, interpersonal violence perpetration, and either adverse outcome, by number of diagnostic categories and gender. Kernel-weighted local polynomial smoothing applied.

Three-quarters of males and females had a psychiatric diagnosis in only one diagnostic category at first discharge (eTable 2 in the Supplementary Material). Figure 1 shows the cumulative incidence for self-harm, interpersonal violence perpetration, and for either adverse outcomes, in the 10 years post-discharge by number of diagnostic categories. The numbers of patients with an adverse outcome at 1 and 10 years are presented in eTable 3 in the Supplementary Material. Risks for all outcomes increased with the number of diagnostic categories.

## Discussion

### Main findings

This national cohort study yielded absolute risk estimates for self-harm and interpersonal violence perpetration among individuals discharged from their first inpatient treatment episode according to gender and psychiatric diagnostic categories. Among males, within a year after first discharge the risk of self-harming was highest among those with substance misuse disorders, whereas for interpersonal violence, individuals diagnosed with early onset behavioral and emotional disorders had the highest absolute risk. The same pattern was observed 10 years post-discharge. For females, absolute risks for self-harm at 1 year and at 10 years were highest among those diagnosed with early onset behavioral and emotional disorders as well as those with substance misuse disorders. Interpersonal violence was most common among those with substance misuse disorders. In both genders, the absolute risks of either internalized or externalized violence within 10 years of first discharge among former inpatients were markedly raised in the riskiest diagnostic categories—substance misuse disorders, personality disorders, and early onset behavioral and emotional disorders. Substance misuse that was comorbid at first discharge with either of these other two high-risk diagnostic categories was the group with the greatest absolute risks. Risk for each examined outcomes also increased with the number of diagnostic categories.

### Comparison with existing evidence

Most previous studies have examined risks of self-harm and violent offending in separate cohorts without the possibility of comparing magnitudes and patterns of risks for different outcomes in the same cohort. Our results do, however, largely concur with results reported from single-outcome cohort studies in the published literature. For instance, Gunnell et al. [7] examined the risk for readmission following self-harm and reported elevated risks among individuals diagnosed with substance misuse disorders and in a miscellaneous “other” diagnostic category. The latter consisted predominantly of personality disorders, concurring with our finding of especially high cumulative incidence values for those two diagnostic groups. Similar patterns were observed by Mellesdal et al. [10]. However, prior to our study, no further examination of gender- and diagnosis-specific risks of self-harm post-discharge has to date been conducted. Direct inter-study comparison of risks across diagnostic categories relies on a similar set of diagnostic codes being used in delineating those groups. In the absence of a uniform approach, comparing our results to those reported by Gunnell et al. [7] and others is only possible to a limited degree.

There is a relatively large body of evidence concerning the risks of dying by suicide following contact with mental health services, and particularly following discharge from an inpatient admission. Using interlinked Danish registry data, as in our study, Nordentoft et al. [16] examined suicide risks following contact with inpatient or outpatient mental health services, with estimates stratified by gender and psychiatric diagnostic category. The highest suicide risks observed were among both men and women diagnosed with bipolar disorder, unipolar affective disorder and schizophrenia, with co-occurring substance misuse and unipolar affective disorders further increasing risk. In another Danish registry study, Qin and Nordentoft [20], focusing specifically on suicide risk following psychiatric admission, found particularly heightened risk among patients diagnosed with affective disorders and for women diagnosed with substance misuse disorders. However, this study also reported that suicide risk associated with affective disorders and schizophrenia spectrum disorders declined relatively quickly after treatment compared to the risk associated with substance misuse disorders. In a large US-based study conducted by Olfson et al. [21], suicide risk within 90 days of discharge was highest among males and females diagnosed with depressive disorders. Individuals with substance misuse disorders had comparatively low suicide rates in this cohort, a finding that contrasts with what we observed. However, the shorter length of follow-up (90 days compared to 1 year in our study) may not have captured the longer-term effects of substance misuse on suicide risk. Direct comparison with these findings is challenging due to our study outcome including both fatal and nonfatal self-harm episodes. However, given that self-harm is the strongest known risk factor for suicide, better understanding the spectrum of fatal and nonfatal self-harming behaviors among discharged inpatients is crucial; reducing risks of self-harm is a key and necessary component in suicide prevention strategies [11].

Exposure to family substance misuse in childhood is a common risk factor for the development of childhood emotional and behavioral disorders [22]. These diagnoses also commonly co-occur with other psychiatric diagnoses, including substance misuse, as well as involvement with the criminal justice system. Therefore, given that the diagnostic categories that we examined were not mutually exclusive, some of the elevated risk observed among the cohort members in our study is likely to partly reflect especially heightened risk among people with a co-occurring diagnosed or undiagnosed substance misuse disorder.

Several other investigations have explored the risk of violent criminal offending, including homicide perpetration, among individuals with a history of inpatient psychiatric treatment or in persons diagnosed with severe mental disorders, typically requiring inpatient treatment [8, 9, 23–25]. Most studies have focused exclusively on a specific subgroup of patients, such as those diagnosed with schizophrenia or other psychoses [8, 9]. In our post-discharge study cohort, the absolute risk of interpersonal violence perpetration was not elevated among persons diagnosed with schizophrenia compared to most other psychiatric diagnoses. However, the absolute risk was still significantly greater than among cohort members who were diagnosed with mood disorders.

### Clinical implications and future research

Patients diagnosed with substance misuse disorders should be offered treatment to address their condition whilst they are still inpatients, irrespective of the reason for their current admission. The positive effect of substance misuse treatment has been shown in several studies [26]. Thus, avoiding delays in receiving effective post-discharge treatments might lead to a reduction in post-discharge risks in this group. Furthermore, given that substance misuse disorders may have a more prolonged effect on increasing self-harm risk [20], the benefits for patients and for services are likely to be substantial. Consideration of the specific needs of patients diagnosed with early onset behavioral and emotional disorders is indicated, particularly given the multitude of challenging and damaging situations likely to have been experienced by this group [22]. Given the high absolute risk for both adverse outcomes among those diagnosed with personality disorders, it is also especially important to put in place suitable interventions and treatment options for this high-risk group.

Recent adverse life events have also been shown to be associated with increased suicide risk after discharge from inpatient treatment [27] and should be considered by clinicians when planning further community-based mental health follow-up. Assessing the environment and social circumstances that patients are discharged into, and arranging appropriate longer-term follow-up, are likely to be particularly important in reducing interpersonal violence risk. Pre-discharge assessment of violence risk should be considered mindfully in relation to potentially complex ethical and legal implications [28]. This assessment should include assessing known predictors, such as history of violence [29], and less commonly used approaches including asking patients about their violent thoughts [30]. However, awareness of the complexity and limitations of violence risk assessment is crucial given that both static and dynamic factors influence likelihood of violent criminality involvement [31]. Further research is therefore needed to provide better guidance for clinicians.

Care planning and coordinated approaches encompassing an array of support services can aid in reducing risks of self-harm and interpersonal violence to protect discharged patients and their families and communities. Therefore, primary and secondary care services should aim to improve the transition from inpatient care, for instance by providing timely at-home care [32]. Care coordinators can enhance this process by supervising and streamlining support in the post-discharge period [33, 34]. Although there is some evidence as regards improving the safety of discharged persons as well as the safety of those in their communities, future research should establish specifically which preventive measures are most effective in reducing risks of self-harm and interpersonal violence in this high-risk group. The positive effects of peer-delivered interventions [35] warrant further research into whether these concepts are suitable for the adverse outcomes examined in this study. Identifying those individuals who are at highest risk for engaging in either of these two harmful behaviors is paramount, and researchers should aim to understand the multiple determinants involved in driving the risk elevations that we and other investigators have observed.

### Strength and limitations

This epidemiological study has revealed informative novel insights into two commonly occurring outcomes among persons discharged from inpatient psychiatric services. It was conducted using high-quality interlinked national registry data with a high degree of completeness and accuracy. The study cohort was large enough to enable analysis with a high degree of statistical power and precision. However, the study had two significant limitations. First, the outcome information that was extracted from these routinely collected administrative datasets was not comprehensive. This is especially pertinent to episodes of nonfatal self-harm that occurred in the community and that did not result in a hospital presentation. Therefore, the registered episodes are overrepresented by more medically serious ones [36]. Interpersonal violence episodes included only criminal offense convictions, thereby excluding police-reported cases and those that went to court and were subsequently dismissed without a conviction, and episodes of interpersonal violence that were not reported to the authorities [1]. Second, for early onset behavioral and emotional disorders, alignment is poor between ICD-8 coding (used between 1969 and 1993) and ICD-10 coding (from 1994) (see eTable 1 in the Supplementary Material). Many individuals who presented with symptoms of these disorders likely will not all have been diagnosed and registered under this category prior to 1994, resulting in misclassification and underreporting of these cases. We were therefore unable to investigate disorder-specific subsets within this broad diagnostic category.

## Conclusion

By providing gender-specific absolute risk estimates according to diagnostic category, our results aid in identifying those individuals at greatest risk of physically harming themselves or other people post-discharge. This informs clinicians to help them to ensure that discharged persons receive the care that they need to keep them and their environment safe. This study has shown the elevated risk for self-harm and interpersonal violence across the spectrum of psychiatric diagnostic categories among people discharged from their first inpatient psychiatric admission. Although risk of self-harm was considerable in the short and longer-term in both genders, risk of violent behavior should also be addressed, particularly among discharged male patients with certain types of disorders. Persons diagnosed with substance misuse disorders, personality disorders and early onset behavioral and emotional disorders were at especially elevated risk for these adverse outcomes. Clinicians must be aware of the greatly raised risks in these specific patient groups and ensure appropriate care and support is offered following discharge.

Patients discharged from inpatient psychiatric care are at greatly elevated risk of perpetrating interpersonal violence, but it is crucial not to further stigmatize this already marginalized group. Even if the absolute risk in these individuals could be reduced to the same level as for persons without a history of psychiatric hospitalization, the total volume and societal burden of interpersonal violence would not be greatly altered. Our findings indicate a need for optimal care at discharge and during the challenging transition that these patients face in resuming their lives in the community, including targeted and individualized inter-agency support and interventions.

## Data Availability

Permission to access our study data is only granted to researchers in the study team. Researchers can apply to access Danish registry data to conduct other studies, in collaboration with experts based at a Danish academic institution.

## Supplementary material

For supplementary material accompanying this paper visit http://doi.org/10.1192/j.eurpsy.2022.2352. click here to view supplementary material
