# Supplementary material for: Absolute risks of self-harm and interpersonal violence by diagnostic category following first discharge from inpatient psychiatric care
Source: Eur Psychiatry. 2023 Jan 18;66(1):e13. doi: 10.1192/j.eurpsy.2022.2352 (PMC9970150; doi:10.1192/j.eurpsy.2022.2352)
Supplement: Supplementary file 1 [file S0924933822023525sup001.docx]

**Online supplementary material**

**eTable 1. Diagnostic classification of psychiatric disorders at first discharge according to the ICD-10-DCR and equivalent ICD-8 ^a^**

| **Diagnosis** | **ICD-10-DCR codes ^b^** | **Equivalent ICD-8 codes** |
| --- | --- | --- |
| Substance misuse disorders | F10-F19 | 291.x9, 294.39, 303.x9, 303.20, 303.28, 303.90, 304.x9 |
| Schizophrenia and related disorders | F20-F29 | 295.x9, 296.89, 297.x9, 298.29-298.99, 299.04, 299.05, 299.09, 301.83 |
| Mood disorders | F30-F39 | 296.x9 (excluding 296.89), 298.09, 298.19, 300.49, 301.19 |
| Anxiety and somatoform disorders | F40-F48 | 300.x9 (excluding 300.49), 305.x9, 305.68, 307.99 |
| Personality disorders | F60 | 301.x9 (excluding 301.19), 301.80, 301.81, 301.82, 301.84 |
| Early onset behavioural and emotional disorders | F90-F98 | 306.x9, 308.0x |

^a^ Between 1969 and 1993 the diagnostic system used was the Danish modification of the International Classification of Diseases, 8th revision (ICD-8)^1^ and, from 1994, the International Classification of Diseases, 10th revision, Diagnostic Criteria for Research (ICD-10-DCR).^2^

^b^ Inclusive of diagnostic categories for which a valid conversion to the ICD-8 code was possible.

**References:**

1. Danish National Board of Health. (1971) Classification of Diseases: Extended Danish-Latin Version of the World Health Organization International Classification of Diseases, 8th Revision, 1965. Copenhagen, Denmark.
2. World Health Organization. (1993). *The ICD-10 Classification of Mental and Behavioural Disorders: Diagnostic Criteria for Research*. Geneva, Switzerland.

**eTable 2. Total numbers of discharged patients by gender and number of psychiatric diagnostic categories ^a^**

| **Number of diagnostic categories** | **Males** | | **Females** | |
| --- | --- | --- | --- | --- |
|  | **N** | **%** | **N** | **%** |
| 1 | 23,201 | 75.3 | 24,707 | 76.9 |
| 2 | 6736 | 21.9 | 6482 | 20.2 |
| 3 or more | 868 | 2.8 | 928 | 2.9 |
|  |  |  |  |  |
| All discharged patients | 30,805 | 100 | 32,117 | 100 |

^a^ Any psychiatric diagnoses not belonging to substance misuse disorders, schizophrenia and related disorder, mood disorders, personality disorders, or early onset behavioural and emotional disorders, were grouped together and counted as an additional category.

**eTable 3. Number of patients with adverse outcomes at 1 year and 10 years post-discharge by number of diagnostic categories and gender**

| **Number of diagnostic categories** | **1 year post-discharge:** | | | **10 years post-discharge:** | | | |
| --- | --- | --- | --- | --- | --- | --- | --- |
|  | **Self-harm** | **Interpersonal violence perpetration** | **Self-harm or interpersonal violence perpetration** | | **Self-harm** | **Interpersonal violence perpetration** | **Self-harm or interpersonal violence perpetration** |
| **Males:** |  |  |  | |  |  |  |
| 1 | 1691 | 868 | 2445 | | 3960 | 3019 | 5992 |
| 2 | 641 | 325 | 905 | | 1352 | 1019 | 2008 |
| 3 or more | 79 | 51 | 125 | | 192 | 160 | 291 |
|  |  |  |  | |  |  |  |
| All discharged patients | 2411 | 1244 | 3475 | | 5504 | 4198 | 8291 |
|  |  |  |  | |  |  |  |
| Matched general population comparison cohort | 1227 | 3688 | 4851 | | 7675 | 16,860 | 22,942 |
|  |  |  |  | |  |  |  |
| **Females:** |  |  |  | |  |  |  |
| 1 | 2396 | 154 | 2511 | | 4740 | 609 | 5034 |
| 2 | 840 | 44 | 873 | | 1564 | 197 | 1650 |
| 3 or more | 151 | 15 | 163 | | 245 | 41 | 269 |
|  |  |  |  | |  |  |  |
| All discharged patients | 3387 | 213 | 3547 | | 6549 | 847 | 6953 |
|  |  |  |  | |  |  |  |
| Matched general population comparison cohort | 1703 | 370 | 2059 | | 8537 | 1814 | 10,022 |
